# Mesonephric adenocarcinoma of the uterine corpus with intracystic growth completely confined to the myometrium: a case report and literature review

**DOI:** 10.1186/s13000-017-0655-y

**Published:** 2017-08-25

**Authors:** Hiroka Ando, Yuko Watanabe, Minori Ogawa, Hiromi Tamura, Tomomi Deguchi, Kayo Ikeda, Mayumi Fujitani, Mitsunori Shioji, Tomoko Tsujie, Reiko Doi, Akinori Wakimoto, Shiro Adachi

**Affiliations:** 1 Departments of Pathology, City Hospital of Toyonaka, Toyonaka, Osaka Japan; 2 Departments of Genecology and Obstetrics, City Hospital of Toyonaka, Toyonaka, Osaka Japan

**Keywords:** Mesonephric adenocarcinoma, Uterine corpus, Intracystic growth

## Abstract

**Background:**

Mesonephric adenocarcinoma (MA) is a rare tumor believed to arise from mesonephric remnants occurring mostly in the uterine cervix and, to a lesser extent, the corpus. Since the first case report of MA in the corpus in 1995, only 16 cases have been reported in the English literature. A recent report suggested that MA originates in Müllerian tissue and exhibits the mesonephric differentiation phenotype.

**Case presentation:**

An asymptomatic 61-year-old woman was referred to our hospital because of elevated levels of tumor markers. Imaging revealed an intramural lesion of the uterine corpus exhibiting fluorodeoxyglucose uptake. A total hysterectomy and bilateral salpingo-oophorectomy were performed. The tumor was completely confined to the corpus wall and was composed of an intracystic bulky component and an invasive component in the myometrial layer. The tumor exhibited a variety of growth patterns, including a characteristic tubular pattern with dense eosinophilic secretion reminiscent of the thyroid, as well as a variety of morphologies, such as acinar, papillary, and ductal structures. The structures were immunoreactive for CK7, vimentin, CD10, calretinin, PAX8, and GATA3 and almost completely negative for ER/PgR. CA125 and CA19–9 antigen expression was also detected.

**Conclusion:**

A case of MA with a unique growth pattern of an intracystic mass within the corpus wall is presented. The histogenesis and differential diagnoses are discussed. The histogenesis of MA is not yet clear. We hypothesize two different pathways involved: 1) direct development from the mesonephric remnants and/or 2) mesonephric transformation of Müllerian adenocarcinoma.

## Background

Mesonephric adenocarcinoma (MA) is a rare tumor of the female genital tract occurring mostly in the uterine cervix. MA is believed to arise from benign mesonephric remnants or hyperplasia located in the lateral walls of the uterine cervix [1]. MA is exceptionally rare in the uterine corpus; only 16 cases have been reported [2–10]. Here we present a case of MA with a unique growth pattern within the myometrial layer of the uterine corpus and provide a review of published cases.

## Case presentation

An asymptomatic 61-year-old woman (gravida 0, reaching menopause in her 40s) was referred to our hospital because of elevated levels of CA125 (389 U/mL; normal range < 35 U/mL) and CA19–9 (785 U/mL; normal range < 37 U/mL). Pelvic magnetic resonance imaging showed a heterogenous mass 7.3 cm in diameter composed of an enhanced solid area and a bloody liquid area, suggesting degenerated leiomyoma or leiomyosarcoma. Endometrial and cervical cytology was performed, which did not indicate any malignant tumors. Because significant uptake of fluorodeoxyglucose was observed in the lesion, a total hysterectomy and bilateral salpingo-oophorectomy were performed. After surgery, the peripheral levels of both tumor markers returned to normal. The patient then underwent adjuvant chemotherapy (paclitaxel and carboplatin) and currently remains free of disease 9 months since the operation.

**Fig. 1:**
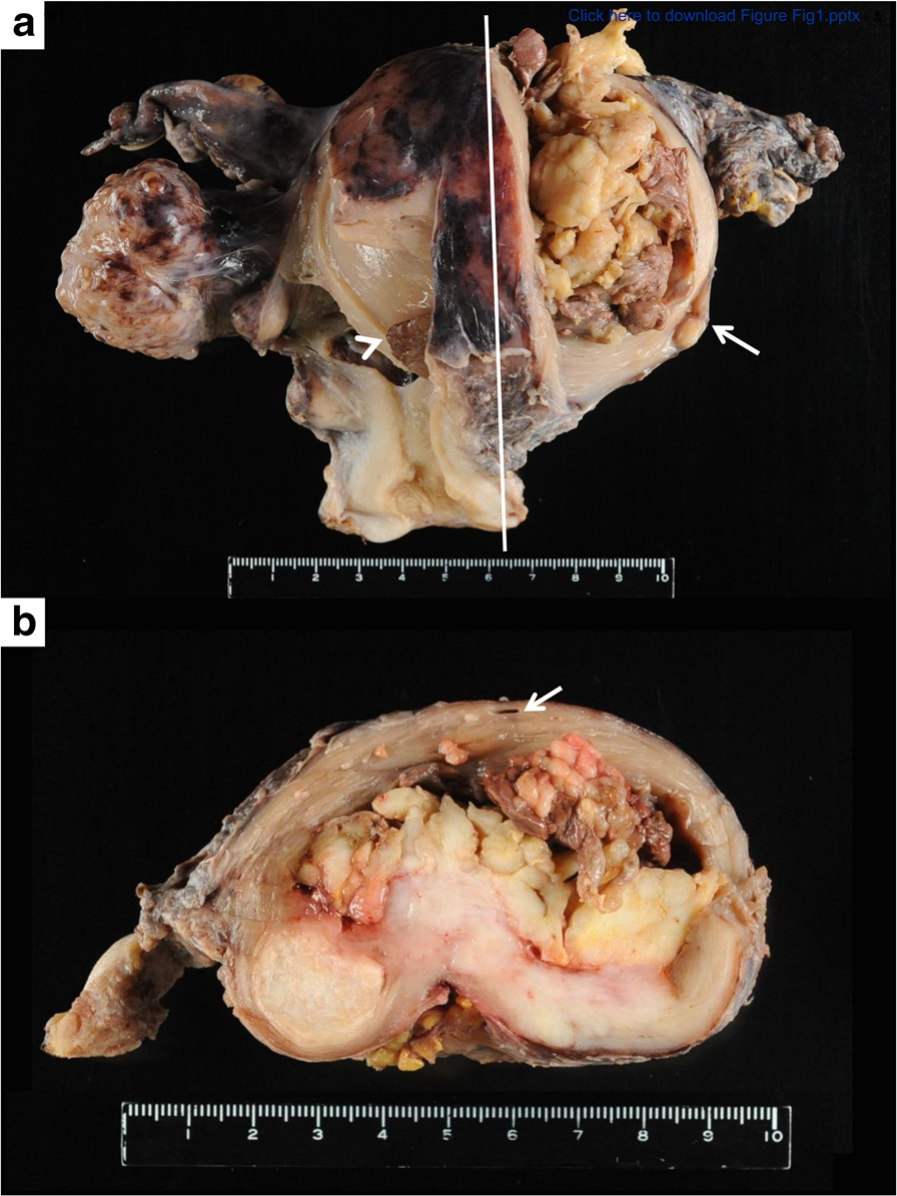
**a** Macroscopic view of the resected specimen. An intramural polypoid tumor protrudes from the incision of the lateral wall of the corpus (*arrow*). The cervical-endocervical canal and corpus cavity are observed to the right of the incision. An endometrial polyp is observed (*arrowhead*). The endometrium and endocervical mucosa are not involved. **b** Sagittal section of the uterus (a cross-section of the line in Fig. 1a). The *arrow* indicates the edge of the endometrial cavity. The yellow-white polypoid tumor appears to be situated in the cyst within the myometrial layer. The tumor invades the posterior wall of the corpus transmurally. A leiomyoma nodule is observed immediately to the left

Macroscopic examination of the resected specimen did not reveal any significant changes in the luminal surface of the uterine cervix, endocervix, endometrium, or peritoneal surface, except for an endometrial polyp and intramural and subserosal myomas. The tumor (8 cm × 6.5 cm) was located in the left-sided posterolateral wall of the corpus and was entirely confined to the myometrium (Fig. 1a). The tumor was comprised of a yellow–white bulky polypoid mass in an intramural cyst containing brownish serous fluid and an invasive lesion in continuum with the intracystic component (Fig. 1b). No abnormalities in the uterine cervix or adnexa were observed.

**Fig. 2:**
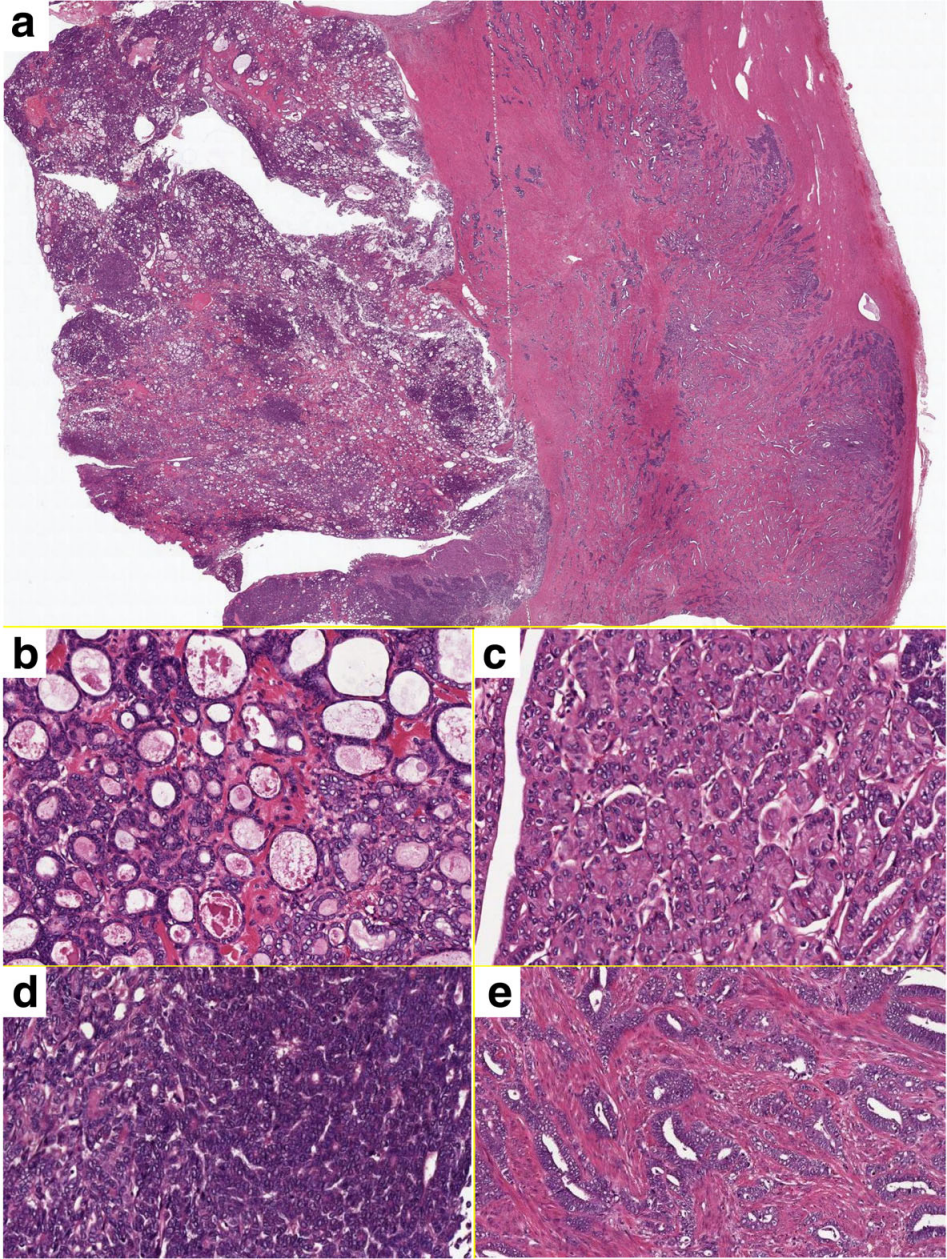
Hematoxylin and eosin staining. **a** Panoramic view of the tumor. The left half of the tissue is the intracystic component, and the right half is the invasive component in the myometrial layer. **b** Relatively uniform tubules containing dense eosinophilic secretion is characteristic. **c** Tumor cells within the eosinophilic cytoplasm are lined in an acinar fashion. **d** Tumor cells show a very high nuclear-cytoplasmic ratio in a solid pattern. **e** Invasive carcinoma in the myometrium. Much higher-grade carcinoma similar to conventional endometrioid adenocarcinoma invades the myometrial layer

The tumor was composed of an intracystic bulky component confined to the intramural cyst and an invasive component in the myometrial layer (Fig. 2a). In the former component, a variety of morphologic patterns, such as tubular, papillary, acinar, and solid growth, were observed. These patterns were admixed but changed abruptly in some areas. The predominant and characteristic growth pattern was a tubular pattern, in which back-to-back, small, round, uniform tubules were lined by cuboidal or flattened cells, and some of their lumens exhibited densely eosinophilic, PAS-positive secretion (Fig. 2b). Acinic and solid patterns were observed at the periphery of the mass neighboring the myometrial wall (Fig. 2c, d). The tumor cells were relatively bland and absent from the nuclear pleomorphism, although occasional mitotic figures were observed. In addition to the abovementioned component at the periphery of the intracystic mass, a more atypical tumor component invaded the myometrial wall (Fig. 2e). This component exhibited a ductal pattern with many mitotic figures and moderate cytologic atypia. No squamoid or mucinous elements were detected. Invasion was observed from the outer-most layer of the cyst to just beneath the peritoneal surface. No tumor necrotic foci were observed in either component.

No neoplastic changes were observed in the cervix, endometrium, or fallopian tubes. Scattered ectopic endometrial tissue was observed in the myometrium. Despite a careful and thorough examination, no mesonephric remnants were identified in the myometrium or uterine cervix.

**Fig. 3:**
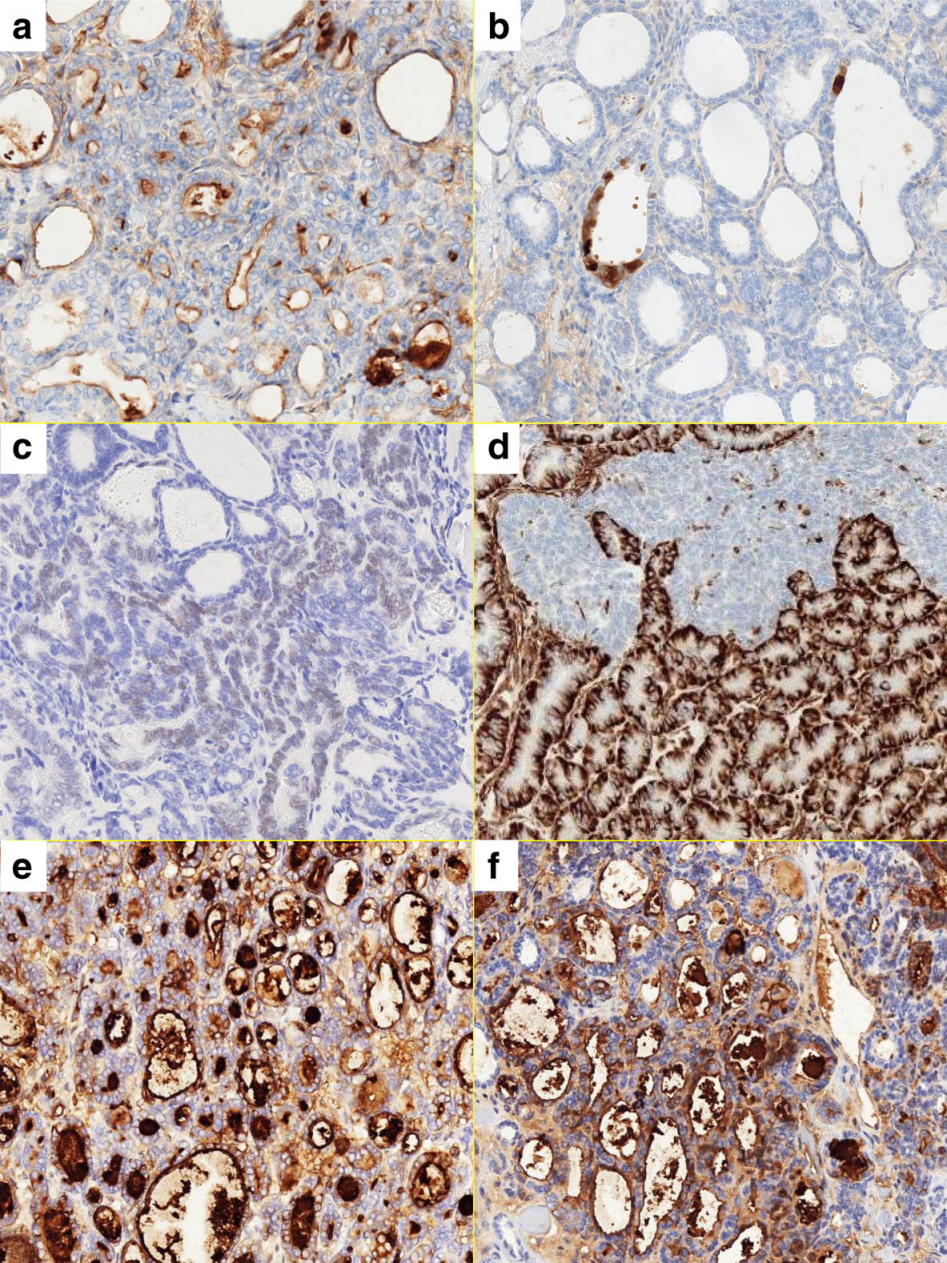
Immunohistochemical staining of the tumor. **a** Luminal expression of CD10 in the neoplastic tubules is observed focally. **b** Focal cytoplasmic and nuclear expression of calretinin. **c** Focal and weak expression of GATA3. **d** Vimentin shows diffuse expression in the acinar pattern but lacks expression in the solid pattern. **e** and **f**, respectively In the tubular pattern, many tumor cells express CA125 and CA19–9

Regardless of the differences in histologic patterns, all neoplastic cells were diffusely positive for CK7 and PAX8 and completely negative for CK20, inhibin, PgR, and WT1 (Table 1). Only a small number of cells (<1%) in the tubular pattern expressed ER and Napsin A; neither was observed in other patterns. Luminal staining of the neoplastic tubules for CD10, nuclear and cytoplasmic staining for calretinin, and nuclear staining for GATA3 were observed focally (Fig. 3a–c). Tumor cells in the tubular and acinar patterns exhibited diffuse expression of vimentin and TTF-1, whereas no expression was observed in those in the solid pattern (Fig. 3d). Focal but intense expression of CA125 and CA19–9 was observed in the tumor regardless of the histologic pattern (Fig. 3e, f). A high labeling index of Ki67 antigen was observed in the solid and ductal patterns in the myometrial layer but was much lower in the tubular and acinar patterns in the cystic component. Significant expression of p53 antigen was not observed in either component.

**Table 1:** Immunohistochemical staining results

Antibody (Manufacture)	Tubular	Acinar	Solid	Ductal
CK7 (Roche)	4+	4+	3+	3+
CK20 (Roche)	0	0	0	0
CD10 (Roche)	4+	0	0	2+
Vimentin (Roche)	4+	4+	0	2+
Calretinin (Roche)	2+	0	0	1+
GATA3 (Nichirei)	2+	0	0	1+
Inhibin (DAKO)	0	0	0	0
ER (Roche)	1+	0	0	0
PR (Roche)	0	0	0	0
p16 (Roche)	2+	2+	2+	4+
PAX8 (BIOCARE)	4+	4+	4+	4+
CA125 (DAKO)	4+	1+	4+	3+
CA19–9 (Roche)	3+	1+	2+	2+
TTF1 (Roche)	4+	4+	0	2+
Napsin (Roche)	1+	0	0	0
WT1 (Roche)	0	0	0	0
Ki67 (Roche)	4%	3%	40%	50%
p53 (Roche)	0	0	0	0

0: 0; 1+: <1% positive cells, 2+: 1–10% positive cells; 3+: 10–50% positive cells; 4+: >50% positive cells; Ki67: Ki67 labeling index

## Discussion

MA is a rare malignancy of the female genital tract occurring mostly in the cervix [11] and rarely in the vagina [12]. Few cases of MA have been reported in the uterine corpus [2–10]. A unique histologic pattern of uniform small tubules containing intraluminal hyaline material is reminiscent of mesonephric remnants [13]. The remnants are sometimes found within the lateral wall of the cervix, mesoovaries, or broad ligaments. Although much more rare, the remnants can also be found in the lateral wall of the vagina or uterine corpus [14]. MA of the cervix or vagina is believed to originate in the mesonephric remnants. However, the question about a real corpus MA has been raised by McFarland et al., who described corpus mesonephric-like adenocarcinomas that appeared to arise in the endometrium and infiltrate into the myometrium [9]. These authors proposed that corpus MA originated from Müllerian tissue and obtained the morphological and immunohistochemical phenotype characteristic of mesonephric differentiation; this tumor is described as a ‘mesonephric-like adenocarcinoma’ rather than MA. In addition to that case, 10 additional cases of corpus MA, including the present tumor, have been reported [2–8, 10]. While one case was believed to be derived from the endometrium, the epicenter of five tumors was in the myometrial layer [5, 6, 8, 10], and three tumors were completely confined to the myometrial layer [2, 3] (Table 2). It is reasonable to conclude that the tumors originated within the myometrial layer in the latter eight cases. Carcinomas rarely occur within the myometrial layer, with most originating from the ectopic endometrial tissue (adenomyosis). Since adenomyosis is common, it is not unusual to detect the lesion, even in cases of MA. In fact, the presence of adenomyosis was described in two cases, including the present case [10]. In these cases, despite a thorough examination, ectopic endometrial tissue was not found in the tumor. Moreover, we did not observe a carcinoma with the morphologically conventional Müllerian phenotype. Therefore, evidence suggesting that these two tumors arose from ectopic endometrial tissue, even in the presence of adenomyosis, is not convincing. In addition, it is important to remember that most MAs that originated within the corpus myometrium were found in the lateral wall [2, 5, 8, 10], where mesonephric remnants were often observed in the cervix and rarely in the vagina and corpus (Table 2). Since both tumors and mesonephric remnants show a predilection for the lateral wall, it is not unreasonable to believe they are related. Because of the lack of reliable evidence, some pathologists propose to call the tumor of the corpus a “mesonephric-like” adenocarcinoma. Nevertheless, we would like to consider that a “mesonephric-like” adenocarcinoma whose epicenter is in the myometrial layer could be diagnosed as MA if the existence of MA in the cervix is confirmed and the morphology is consistent with that of MA. The cases presented by McFarland et al. are of great interest [9]. In their uterine cases, all the tumors predominantly involved the endometrium and appeared to arise there with subsequent invasion into the myometrium. Therefore, they suggested the intriguing possibility that MAs do not actually arise from mesonephric remnants, but could arise from Müllerian structures and differentiate along a mesonephric pathway. However, based on the morphological features and immunophenotype, they believe these tumors to represent MA. When taking into consideration the description by McFarland et al., it is reasonable to believe that MA can originate in the uterine corpus in two ways: 1) direct development from the mesonephric remnants of the corpus wall and 2) mesonephric differentiation of a Müllerian adenocarcinoma originating from the endometrium, as proposed by McFarland et al.

**Table 2:** Reported cases of mesonephric adenocarcinoma in the uterine corpus

Case	Study	Age	Size(cm)	Mesonephric remnant	Location	Involvement of END	CA125	CA19–9
1	Yamamoto et al. [2]	58	14	Cervix	MYO (left lateral)	-	Normal	Normal
2	Ordi et al. [3]	33	8 × 6	-	MYO	-	47 IU/mL	NA
3	Bagué et al. [4]	37	3.5	-	END	+	NA	NA
4	Marquette et al. [5]	81	3.7 × 3.0 × 2.3	NA	MYO (right lateral)	+	55 kU/mL	NA
5	Wani et al. [6]	73	8 × 7	-	MYO	+	Normal	Normal
6	Kenny et al. [7]	NA	NA	cervix	corpus	NA	NA	NA
7	Wu et al. [8]	55	3.5 × 2.5 × 2.0	NA	MYO, endocervix, (lateral)	+	163.8 U/mL	193.6 U/mL
8	Wu et al. [8]	62	8 × 7 × 3	NA	MYO	NA	Normal	Normal
9–15	McFarland et al. [9]	NA	NA	-	END, subsequent to MYO	+	NA	NA
16	Kim et al. [10]	66	2.5 × 2.0	-	MYO (left anterolateral)	+	Normal	NA
17	Ando et al. (present case)	61	8 × 6.5	-	MYO (left posterolateral)	-	389 U/mL	785 U/mL

*END* endometrium, *MYO* myometrium of the uterine corpus, *NA* not available

MA typically exhibits a variety of architectural growth patterns, even within the same tumor [13]. Although this admixture of growth patterns is a somewhat characteristic feature and can be useful for diagnosis, it may also result in diagnostic issues and mimicry of other neoplasms. The differential diagnosis of MA of the uterus includes endometrioid carcinoma, serous adenocarcinoma, and clear cell carcinoma. The distinction of MA from endometrioid carcinoma and serous adenocarcinoma may be straightforward when the characteristic feature of relatively uniform small tubules containing intraluminal hyaline material, as well as a variety of growth patterns, is found in the tumor. The absence of squamous metaplasia, nuclear stratification, and ER/PgR expression may exclude the diagnosis of endometrioid carcinoma [6]. A similar appearance of uniform small tubules containing intraluminal eosinophilic material is sometimes observed in clear cell carcinoma. Clear cell carcinoma comprises higher-grade atypical tumor cells, even in the uniform small tubules, whereas MA comprises relatively bland-looking tumor cells. A Hobnail-like appearance of the tumor cells and hyalinization of the stroma can also aid in the differential diagnosis. Immunohistochemistry may also be helpful. CD10 and calretinin have been commonly used for supporting mesonephric differentiation, and recently GATA3 has been shown to be a useful marker for mesonephric lesions in the lower female genital tract [15]. However, neither CD10 nor calretinin is highly sensitive or specific for mesonephric differentiation, and GATA3 staining has great variability in both intensity and extent. In addition, weak reactivity of GATA3 has been observed in 7% of endometrial carcinomas [16]. Therefore, at this time, there are no antibodies that distinguish MA from Müllerian carcinomas.

Compared with previously reported cases, the present tumor showed two unique features. The first was its unique gross appearance. Most of the tumor component was present in the cyst within the myometrial layer. There have been only two cases, including ours, that have exhibited an intracystic growth pattern. Yamamoto et al. described a tumor with large cyst formation containing brownish fluids, as seen in the present case [2]. This growth pattern has not been described in cervical MA. The other feature is the possible higher-grade transformation in the same tumor. The tumor is comprised of two different geographical and biological components: 1) the intracystic component composed of low-grade carcinoma with typical MA histology and 2) a higher-grade invasive component within the myometrial layer that merges with the intracystic component. The biological differences in the components correspond to differences in their cytological atypia and Ki67 labeling indices. It is possible that the tumor described in the present case arose as the former type of MA, obtained higher malignant potential, and transformed into adenocarcinoma, invading the myometrium.

The present patient was referred to our hospital because of elevated serum levels of CA125 and CA19–9. Immunohistochemical analysis revealed that the tumor cells expressed both markers, and their concentrations in the peripheral blood returned to normal ranges after tumor removal. Several case reports have described an elevated level of CA125 in MA patients [3, 5, 8], whereas only two case reports have described an elevated level of CA19–9 [8]. This report is the first to show expression of CA19–9 in MA by immunohistochemistry.

It is interesting to note that the first case report of MA in the uterine corpus was not published until 1995. It is also surprising that only 17 cases, including the present case, have been recorded thus far [2–10]. It is possible that several cases may have been diagnosed as Müllerian adenocarcinoma. It is no wonder that some pathologists prefer the term mesonephric-like adenocarcinoma. To clarify the histogenesis and biological behavior, it is important to collect a sufficient number of MA cases for clinicopathological and molecular verification by raising awareness of the presence and characteristic histological features of MA in the uterine corpus.

## Conclusion

We described a case of MA with a unique gross appearance of an intracystic tumor completely confined within the corpus myometrium. While the histogenesis of MA has not yet been confirmed in the uterine corpus, we propose two different pathways by which MA arises in the uterine corpus: 1) direct development from the mesonephric remnants and/or 2) mesonephric transformation of Müllerian adenocarcinoma.

## Abbreviations

MA: Mesoneophric adenocarcinoma
